# Complex post-traumatic stress disorder and post-migration living difficulties in traumatised refugees and asylum seekers: the role of language acquisition and barriers

**DOI:** 10.1080/20008198.2021.2001190

**Published:** 2021-12-07

**Authors:** Jennifer Schiess-Jokanovic, Matthias Knefel, Viktoria Kantor, Dina Weindl, Ingo Schäfer, Brigitte Lueger-Schuster

**Affiliations:** a Department of Clinical and Health Psychology, Faculty of Psychology, University of Vienna, Vienna, Austria; b Department of Psychiatry and Psychotherapy, University Medical Centre Hamburg-Eppendorf, Hamburg, Germany

**Keywords:** Complex post-traumatic stress disorder (CPTSD), refugees, post-migration living difficulties (PMLDs), post-migration stress, language acquisition, childhood trauma, Trastorno de estrés postraumático complejo (TEPT-C), refugiados, dificultades de vida post-migración (DVPM), estrés post-migración, adquisición del lenguaje, trauma infantil, 复杂性创伤后应激障碍 (CPTSD), 难民, 移民后生活困难 (PMLDs), 移民后应激, 语言习得, 童年创伤

## Abstract

**Background**: Numerous traumatic experiences and post-migration living difficulties (PMLDs) increase the risk of developing symptoms of complex post-traumatic stress disorder (CPTSD) among Afghan refugees and asylum seekers, living in Austria. Research has repeatedly associated higher levels of CPTSD with higher levels of PMLDs. Summarizing PMLDs into empirically derived factors might facilitate a further understanding of their interaction with symptom presentation within distinct clusters of CPTSD.

**Objective**: The current study aimed to investigate homogeneous subgroups of ICD-11 CPTSD and their association with demographic variables, traumatic experiences, and empirically derived factors of PMLDs.

**Method**: Within a randomized controlled trail (RCT) CPTSD, PMLDs, and traumatic experiences were assessed in a sample of 93 treatment-seeking Afghan refugees and asylum seekers through a fully structured face-to-face and interpreter-assisted interview using the ITQ, the PMLDC, and a trauma checklist. Underlying clusters of CPTSD, superior factors of PMLDs, and their associations were investigated.

**Results**: In total, 19.4% of the sample met the diagnostic criteria for PTSD and 49.5% for CPTSD. We identified a 2-cluster solution consisting of two distinct subgroups as best fitting: (1) a CPTSD cluster and (2) a PTSD cluster. The multitude of PMLDs was summarized into four superior factors. CPTSD cluster membership was associated with childhood potentially traumatic experience types, and one of four PMLD factors, namely ‘language acquisition & barriers’.

**Conclusions**: The results suggest that not PMLDs in general, but rather specific types of PMLDs, are associated with CPTSD. An assumed bidirectional relationship between these PMLD factors and CPTSD symptoms might lead to a downward spiral of increasing distress, and could be considered in treatment strategies.

## HIGHLIGHTS

- Two homogeneous subgroups of CPTSD symptoms were identified (CPTSD, PTSD).
- Only the PMLD factor ‘language acquisition & barriers’, and childhood potentially traumatic experience types were associated with the CPTSD cluster.

## Abstract (es)

**Antecedentes:** Numerosas experiencias traumáticas y dificultades de vida post-migración (DVPM) aumentan el riesgo de desarrollar síntomas de trastorno de estrés postraumático complejo (TEPT-C) entre los refugiados y solicitantes de asilo afganos que viven en Austria. La investigación ha asociado repetidamente niveles más altos de TEPT-C con niveles más altos de DVPM. Resumir las DVPM en factores derivados empíricamente podría facilitar una mayor comprensión de su interacción con la presentación de síntomas dentro de distintos grupos de TEPT-C.

**Objetivo:** El presente estudio tuvo como objetivo investigar subgrupos homogéneos de TEPT-C según la CIE-11 y su asociación con variables demográficas, experiencias traumáticas y factores derivados empíricamente de DVPM.

**Método:** Dentro de un estudio controlado aleatorizado (ECA), se evaluaron TEPT-C, DVPM y experiencias traumáticas en una muestra de 93 pacientes, refugiados afganos y solicitantes de asilo, a través de una entrevista cara a cara totalmente estructurada y asistida por un intérprete utilizando el ITQ, el PMLDC y una lista de verificación de traumas. Se investigaron los grupos subyacentes de TEPT-C, los factores superiores de DVPM y sus asociaciones.

**Resultados:** En total, el 19,4% de la muestra cumplió los criterios de diagnóstico de TEPT y el 49,5% de TEPT-C. Identificamos 2 grupos que constan de dos subgrupos distintos: (1) un grupo de TEPTC y (2) un grupo de TEPT. Las distintas DVPM se resumieron en cuatro factores superiores. La pertenencia al grupo de TEPT-C se asoció con tipos de experiencias potencialmente traumáticas en la infancia y uno de los cuatro factores de DVPM, denominada, ‘adquisición y barreras del lenguaje’.

**Conclusiones:** Los resultados sugieren que no las DVPM en general, sino los tipos específicos de DVPM, están asociados con el TEPT-C. Una supuesta relación bidireccional entre estos factores de DVPM y los síntomas de TEPT-C podría conducir a una espiral de angustia creciente, y podría considerarse en las estrategias de tratamiento.

## Abstract (zh)

**背景:** 许多创伤经历和移民后生活困难 (PMLD) 增加了居住在奥地利的阿富汗难民和寻求庇护者出现复杂性创伤后应激障碍 (CPTSD) 症状的风险。研究反复将更高水平的 CPTSD 与更高水平的 PMLD 联系起来。将 PMLD 归纳为经历衍生因素可能有助于进一步了解其与不同 CPTSD 簇中症状表现的相互作用。

**目的:** 本研究旨在考查 ICD-11 CPTSD 的同质亚组及其与人口统计学变量, 创伤经历和 PMLD 经历衍生因素的关联。

**方法:** 在一项随机对照试验 (RCT) 中, 通过一个使用 ITQ 的完全结构化的面对面和口译辅助访谈, PMLDC 和创伤检查表, 对一个 93 名寻求治疗的阿富汗难民和寻求庇护者样本中进行了 CPTSD, PMLD 和创伤经历评估。考查了 CPTSD 的潜在簇, PMLD 的高阶因素及其关联。

**结果:** 共有19.4%的样本符合PTSD诊断标准, 49.5%的样本符合CPTSD诊断标准。我们确定了一个由两个不同亚组组成的 2 簇解决方案作为最佳拟合: (1) CPTSD 簇和 (2) PTSD 簇。众多 PMLD 被总结为四个高阶因素。 CPTSD 簇所属与童年潜在创伤经历类型以及四个 PMLD 因素之一有关, 即‘语言习得和障碍’。

**结论:** 结果表明, 不是一般的 PMLD, 而是特定类型的 PMLD, 与 CPTSD 相关。这些 PMLD 因素和 CPTSD 症状之间假设的双向关系可能导致精神痛苦增加的恶性循环, 并且可以在治疗策略中加以考虑。

## 1. Introduction

Since the 1980s, ongoing and renewed conflicts, human rights violations, and unstable security conditions have forced numerous individuals to flee from Afghanistan. As the third most common country of origin, the UN Refugee Agency (UNHCR) recorded around 2.7 million Afghan refugees recognized under international law, and 0.3 million asylum seekers currently awaiting legal recognition, in 2019 (UNHCR, 2020). Compared to refugee populations from other countries of origin in Austria, Afghans report particularly low health (Georges, Buber-Ennser, Rengs, Kohlenberger, & Doblhammer, 2021). Prolonged, repeated, and/or interpersonal traumatic experiences in the country of origin or during flight are highly prevalent among refugees (Bogic, Njoku, & Priebe, 2015), with between 40 and 90% reporting at least one traumatic experience, depending on the sample and location (Scoglio & Salhi, 2020). Thus the diagnosis of complex post-traumatic stress disorder (CPTSD), introduced in the ICD-11, is especially suitable to capture the more far-reaching psychological consequences following complex trauma. In addition to traumatic experiences, it is important to consider post-migration living difficulties (PMLDs) in Austria in order to understand better the mental health problems in refugees. However, it is not yet clear how CPTSD and PMLDs relate to one another.

### 1.1. Post-migration living difficulties

Traumatic exposure has been shown to severely impact mental health (Bogic et al., 2015; Cloitre et al., 2009). In recent years, increasing attention has been paid to the influence of the multiple, heterogeneous stressors associated with facing a foreign country, language, and culture following migration on mental health (Hynie, 2018; Li, Liddell, & Nickerson, 2016). The term post-migration living difficulties (PMLDs) subsumes various different constructs, including interpersonal stressors (e.g. discrimination, social isolation), emotional stress (e.g. feelings of loneliness), stressors associated with the asylum process (e.g. work permit, insecure visa), or migration-related aspects (e.g. family separation, language barriers) (Li et al., 2016; Schweitzer, Melville, Steel, & Lacherez, 2006). Despite this heterogeneity, however, most previous studies used single sum scores to capture a potentially common entity (Silove et al., 2018), or selected specific individual PMLDs (Liddell et al., 2019) to investigate their association with mental health problems. So far, these studies have shown that while stress and feelings of anxiety may be part of a normal response associated with PMLDs, chronic exposure to PMLDs increases the risk of developing serious mental health problems at least to the same extent as traumatic experiences (Bogic et al., 2015; Li et al., 2016).

Different PMLDs might have distinct effects on mental health, and an empirical subdivision would therefore allow for a more differentiated investigation of the effects. To date, there have only been a small number of attempts to categorize the high number of PMLDs and to analyse their common effects without merely using a single sum score. Silove (1998)first subsumed the individual PMLDs into five underlying factors using exploratory factor analysis. Further efforts to categorize the total number of PMLDs resulted in partly similar domains depending on the sample, host country, and PMLDs assessed, with mostly separate groups concerning the asylum procedure, socio-economic living conditions, discrimination, and family concerns (Laban, Gernaat, Komproe, Van Der Tweel, & de Jong, 2005; von Haumeder, Ghafoori, & Retailleau, 2019). Due to the small number of studies in this regard, and the influence of regional differences, no consensus on the subdivision of PMLDs has yet been reached. Based on the aforementioned assumption that the multitude of heterogeneous PMLDs could have different impacts on mental health, researchers have emphasized the importance of further investigating the effects of different types of PMLD on mental health (Hou et al., 2019).

### 1.2. Complex post-traumatic stress disorder

In order to better capture the complex consequences of traumatic experiences, the diagnosis of complex post-traumatic stress disorder (CPTSD) was introduced in the ICD-11, comprising in total six symptom clusters. Three symptom clusters (re-experiencing, avoidance, sense of current threat) are shared with post-traumatic stress disorder (PTSD) and the remaining three symptom clusters are linked to disturbances in self-organization (DSO; affect dysregulation, negative self-concept, difficulties in interpersonal relationships). In addition, both diagnoses require functional impairment to be considered fulfilled (WHO, 2019).

A growing body of research has examined groups of trauma survivors characterized by different CPTSD symptom presentations as well as various variables associated with these different presentations (Brewin et al., 2017). While several studies investigated CPTSD in various samples (Knefel, Garvert, Cloitre, & Lueger-Schuster, 2015), only a few of them took into account refugee samples (e.g. Barbieri et al., 2019). Nonetheless, the initial evidence supports the suitability of the diagnosis of CPTSD in refugee populations and indicates cross-cultural validity (Hyland et al., 2018). Most refugee studies identified three homogeneous subgroups including CPTSD, PTSD, and a low symptom group (Hyland et al., 2018), or four subgroups with an additional group characterized by elevated affective dysregulation (Liddell et al., 2019). A lower number of homogeneous subgroups of individuals with CPTSD has rarely been identified (Barbieri et al., 2019; Palic et al., 2016). Investigations of demographic variables associated with membership of the CPTSD subgroup have yielded inconsistent findings. While some studies reported no associations with demographic variables (Barbieri et al., 2019; Hyland et al., 2018), others found that variables such as female gender (Liddell et al., 2019), lower educational attainment, or living alone (Perkonigg et al., 2015) were associated with membership of the CPTSD subgroup. Further results indicated associations of CPTSD with functional impairment (Hyland et al., 2018; Palic et al., 2016; Perkonigg et al., 2015), cumulative trauma (Liddell et al., 2019), and a higher number of comorbidities (Murphy, Elklit, Dokkedahl, & Shevlin, 2016). While the characteristics of CPTSD subgroups in refugee samples have been sparsely investigated, to the best of our knowledge, there is no study with a focus on the even more burdened treatment-seeking population of refugees from Afghanistan who have a long history of war and violence.

### 1.3. Associations between CPTSD and PMLDs

While PMLDs overall have been found to predict CPTSD symptom severity (Hecker, Huber, Maier, & Maercker, 2018), the specific contribution of different aspects of PMLDs to CPTSD symptom presentation has not yet been investigated. A study using the aforementioned single sum score of PMLDs found first evidence suggesting an association between elevated CPTSD and an overall higher degree of PMLDs in refugees (Tay et al., 2018). Another study also found a positive relationship of individual PMLDs, such as insecure asylum status, with CPTSD in refugees and asylum seekers (Liddell et al., 2019). As the ICD-11 CPTSD is a rather new diagnosis, studies utilizing this classification are only just beginning to emerge, although there is more research suggesting an association between individual PMLDs and the symptoms of PTSD. For example, language learning difficulties (Kartal, Alkemade, & Kiropoulos, 2019; Söndergaard & Theorell, 2004), occupational problems, and difficulties in accessing social and health services (Hou et al., 2019) were found to be associated with increased PTSD symptoms. The results of a mediator analysis conducted within a meta-analysis indicated a positive association between prior traumatic experiences and PTSD, which was mediated by different forms of PMLDs such as interpersonal daily stressors (Hou et al., 2019).

Taken together, to date, findings on CPTSD and its association with PMLDs in refugees are scarce, and the assumed complex and possibly varying relationships of different types of PMLDs with CPTSD remain unclear (Barbieri et al., 2020). We aimed to contribute to a better understanding of the patterns of CPTSD symptom presentation and their associations with traumatic experiences, distinct types of PMLDs, and demographic variables in treatment-seeking Afghan refugees and asylum seekers. Specifically, the present study aimed to (1) examine emerging subgroups of CPTSD symptoms, (2) explore the factor structure of PMLDs, and (3) investigate the associations of different CPTSD symptom subgroups with demographic variables, traumatic experiences, and PMLD factors.

## 2. Methods

Data were collected as part of the baseline assessment of a randomized controlled trial (RCT; PIAAS-Study; Knefel et al., 2020) between July 2019 and December 2020. All participants provided full informed consent, and the study was approved by the ethics committee of the University of Vienna (reference numbers: 00356 and 00445).

### 2.1. Participants

**Figure 1.**
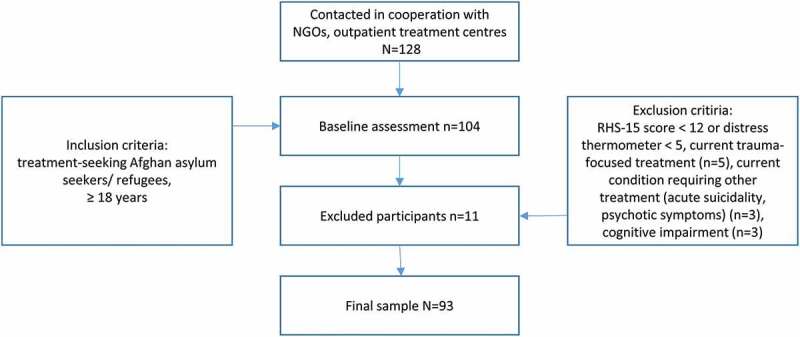
Participants flow chart

The final sample comprised 93 treatment-seeking, Dari-speaking adult Afghan refugees or asylum seekers in Austria. Inclusion criteria were: (a) psychological treatment-seeking in specific institutions (b) increased mental distress based on a screening questionnaire (RHS-15). Exclusion criteria were (a) a current condition that impeded participation and required other treatment (severe mental disorder such as psychotic disorders or substance dependence, acute suicidality), (b) severe cognitive impairment or (c) current psychological treatment (Figure 1). Due to the low literacy rate, the conditions of participation were thoroughly discussed with each participant before consent was obtained. Participants were reimbursed for travel expenses. Sample size calculations and power analysis were conducted for the RCT and can be found in the published study protocol (Knefel et al., 2020). Sample characteristics are reported in Table 1.

**Table 1.** Sociodemographic characteristics

Baseline Characteristic	Total (*N* = 93)
	*n* (%/SD)
**Gender (%)**	
Female	42 (45.2)
Male	51 (54.8)
**Age (SD)**	34.77 (13.84)
**Marital status**	
Single	37 (39.8)
Married/Cohabiting	48 (51.7)
Divorced/widowed	7 (7.6)
**Highest educational achievement (%)**	
No formal education	35 (37.6)
Elementary school	21 (22.6)
Secondary school	16 (17.2)
High school	16 (17.2)
University	4 (4.3)
**Employment(%)**	
Employed or in training	7 (7.5)
Student	11 (11.8)
Unemployed without work permit	21 (22.6)
Unemployed	36 (38.7)
Unable to work/ permanently ill	2 (2.2)
Retired	2 (2.2)
Unpaid housework, childcare	2 (2.2)
Other (e.g. community service)	7 (7.5)
**Asylum Status (%)**	
Granted asylum	40 (43.0)
Subsidiary protection	18 (19.4)
Asylum seeker/ asylum seeker appealing rejection	27 (29.0)
Austrian citizenship	3 (3.2)
Other	4 (4.3)

Note: Numbers may not sum to total *N* and percentages may not sum to 100 due to missing values.

### 2.2. Measures

Sociodemographic data were assessed at the baseline assessment, including general and specific information relevant to the research question.

German and Dari versions of the International Trauma Questionnaire (ITQ), Post-Migration Living Difficulties Checklist (PMLDC) and Harvard Trauma Questionnaire (HTQ) were provided. The assessment was conducted as a fully structured interview with a trained psychologist and a specifically trained interpreter. Additionally, all Likert scales were displayed visually to better represent the gradations in the response options.

The ITQ (Cloitre et al., 2018) measures six symptom clusters of CPTSD and additionally functional impairment in different contexts using 18 items. Participants were asked to indicate how often they had experienced post-traumatic symptoms in the last month on a 5-point Likert scale from ‘not at all’ (0) to ‘extremely’ (4). The criteria for the symptom cluster were considered to be fulfilled if at least one of the two respective items resulted in a value equal to or higher than 2. To meet the CPTSD criteria, all six symptom clusters had to be met and functional impairment due to PTSD and DSO had to be present. Initial validations of the ITQ have shown good psychometric properties. The Cronbach’s alpha coefficient in the present study was .89.

The PMLD Checklist is a self-report questionnaire used to assess a wide range of PMLDs in refugees and asylum seekers (Silove, Sinnerbrink, Field, Manicavasagar, & Steel, 1997). The final version was adapted for Austria and contains 26 items (Knefel et al., 2020). Participants were asked to record the frequency of experiencing the different PMLDs in the last month on a 5-point Likert scale from ‘not at all’ (0) to ‘extremely’ (4). For an overview of the items and adaptations, see Table 2. The Cronbach’s alpha coefficient in the present study was found to be .77.

**Table 2.** Regularized exploratory factor analysis

Items*How often was the problem listed below true for you?*	Factor	I	II	III	IV
13. Not having enough money to buy food, necessary clothing, or pay rent	DS	.**76**	−.02	−.12	.09
14. Difficulties in obtaining financial support	DS	.**68**	−.03	−.01	.15
2. Discrimination	DS	.**47**	−.01	−.14	−.23
17. Difficulties in obtaining adequate housing	DS	.**46**	.12	.25	−.09
22. Dependence on others due to language (loss of autonomy)*	LAB	−.05	.**72**	−.10	.13
1.Communication difficulties	LAB	−.26	.**63**	−.13	.05
24. Difficulties in understanding bureaucratic processes in Austria*	LAB	.18	.**66**	.11	−.30
16. Difficulties learning German	LAB	.00	.**55**	.02	.07
19. No contact with family and friends in the country of origin*	LAB	.19	.**41**	.16	.13
10. No recognition as a refugee	RI	−.09	−.22	.**76**	.01
11. Fear of future deportation to the homeland	RI	−.04	.16	.**69**	.08
21. Negative media reports about Afghan fellow citizens in Austria*	RI	.21	.10	.**36**	.05
4. Family separation	FC	−.02	.01	.30	.**60**
26. Homesickness*	FC	.20	.02	.00	.**58**
5. Concern for family members remaining in the home country or living far away	FC	.09	−.03	−.06	.**47**
3. Conflicts with own or other ethnic groups in Austria	X	.**34**	−.05	.20	.18
6. Impossibility to travel home in case of emergency	X	.12	.01	.06	.32
7. Difficulties with work (e.g.: work permit, working conditions)	X	.13	.08	.07	.25
8. Difficulties with official channels (e.g.: interview with asylum agency)	X	.**35**	.13	.**30**	−.27
9. Conflicts with authorities	X	.**30**	−0.05	.15	**−.35**
12. Worries about not receiving medical support or treatment for health problems	X	.29	.**36**	−.01	−.01
15. Loneliness, boredom or isolation	X	.15	.16	.**30**	.28
18. Family pressure, expectations which cannot be fulfilled*	X	.**33**	.03	−.18	.**39**
20. Stressful media reports and social media content*	X	.08	.15	.25	.27
23. Stigmatization due to origin*	X	.**33**	.04	.29	.08
25. Different social norms than in the country of origin*	X	.19	.03	.13	−.10

Note: *N* = 93. * Items added in the adapted checklist. The extraction method was regularized least squares estimation with an oblimin rotation. Factor loadings above .30 are in bold. X = excluded items. Missing data were handled with predictive mean matching was used.

Potentially traumatic experiences were assessed with an adapted version of the Trauma Checklist of the Harvard Trauma Questionnaire (HTQ) (Mollica et al., 1992) including 29 dichotomous items. Participants were asked to indicate whether they had experienced or witnessed the events personally. In addition, a specifier recorded when the trauma was experienced (childhood, adulthood, or both). The questionnaire was developed for refugees and has been validated in various studies (Kleijn, Hovens, & Rodenburg, 2001).

## 3. Analysis

The analytical strategy for the current study included three steps, corresponding to the three study aims.

First, a cluster analysis was conducted for all 20 imputed data sets following the recommended steps (Basagaña, Barrera-Gómez, Benet, Antó, & Garcia-Aymerich, 2013) to cluster the six CPTSD symptom domains into meaningful, mutually exclusive subgroups based on similarities among the data. The resulting homogeneous subgroups exhibit high external (between-group) heterogeneity and internal (within-group) homogeneity (Balijepally, Mangalaraj, & Iyengar, 2011). Cluster analysis allows quantification of structural features of observations and is useful for simplifying data, describing taxonomies, and identifying relationships. The R package ‘NbClust’ was used to determine the relevant number of clusters in a dataset by evaluating 26 different indices, varying cluster size and distance measures (Charrad, Ghazzali, Boiteau, & Niknafs, 2014). After determining the relevant number of clusters, the k-means algorithm assigns each observation to exactly one of the k clusters. The k-means method is a centroid-based clustering and divides the observations into k clusters based on an iterative clustering algorithm. It was performed with the package ‘stats’ in R (R Core Team, 2021). Further analysis of the cluster characteristics were used for cluster validation.

Second, regularized exploratory factor analysis (REFA) was applied to the first with predictive mean matching imputed data set (McNeish, 2016) to summarize the individual PMLDC items into a smaller number of superior factors. This method is especially suitable for small samples and can thus provide stable factor-loading estimates (Jung & Lee, 2011). We used the R package ‘fungible’ (Waller, 2021). In contrast to confirmatory factor analysis, EFA does not make any prior assumptions about the number of factors and the relevant relationships between the variables; we chose this approach because it would not have been possible to proceed in a theory-based manner based on the current state of research. Due to non-normal distribution of the data, a principal factor method was chosen as recommended (Costello & Osborne, 2005), with an oblique (oblimin, direct) rotation. The number of factors to retain was based on several criteria: (1) a visual examination of the scree plot; (2) parallel analysis of Velicer’s minimum average partial (MAP) test (O’Connor, 2000), and (3) considerations regarding the meaning and interpretability of the factor model. Items that loaded more than .30 on a primary factor and did not have any cross-loadings (O’Connor, 2000) with less than .2 difference (Gaskin, 2015) were summarized into sum scores.

Third, to examine the associations between CPTSD clusters and various variables (childhood potentially traumatic experience (PTE) types, adulthood PTE types, PMLD factors) which are assumed to characterize cluster membership, multivariate analysis of variance (MANOVA) and subsequent *p*-value pooling was applied (Finch, 2016). Pillai’s Trace was applied as a multivariate statistic because this estimator is more robust and less sensitive to violations of multivariate normality. As a nonparametric alternative, multiple imputation student’s *t* t-tests were applied for post-hoc analysis. The association between CPTSD clusters and gender was investigated with χ^2^ tests. The MANOVA and post-hoc tests were calculated using ‘stats’ package in R (R Core Team, 2021).

### 3.1. Missing data

In the six symptom clusters for CPTSD and functional impairment, a total of 3.3% (3.2–4.3%) single data points were missing, stemming from four incomplete cases. For the PMLDC, 3.6% (3.2–5.4%) missing values were found from 10 incomplete cases. Due to a more than 50% high proportion of missing values, three cases were deleted completely. Missing values were estimated by multiple imputation (*m* = 20, method = ‘predictive mean matching’) using Fully Conditional Specifications (FCS; Van Buuren & Groothuis-Oudshoorn, 2011), an algorithm in the R package ‘mice’.

**Results**

This section is organized according to the three aims of the study.

### 3.2. CPTSD symptom patterns

**Figure 2.**
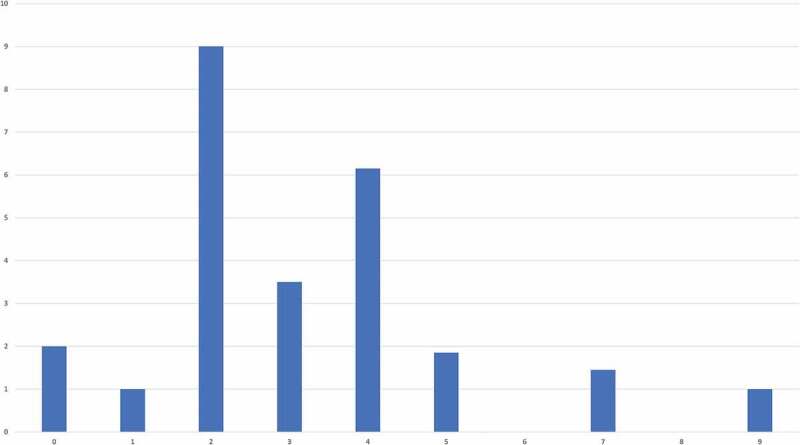
NbClust’s optimal number of clusters

Note: Average best number of clusters determined with NbClust by evaluating 26 indices. A total of *m*=20 imputed data sets were considered.

Cluster solutions from zero to nine were evaluated using 26 fit indices. Most fit indices favoured a two cluster solution (Figure 2).

**Figure 3.**
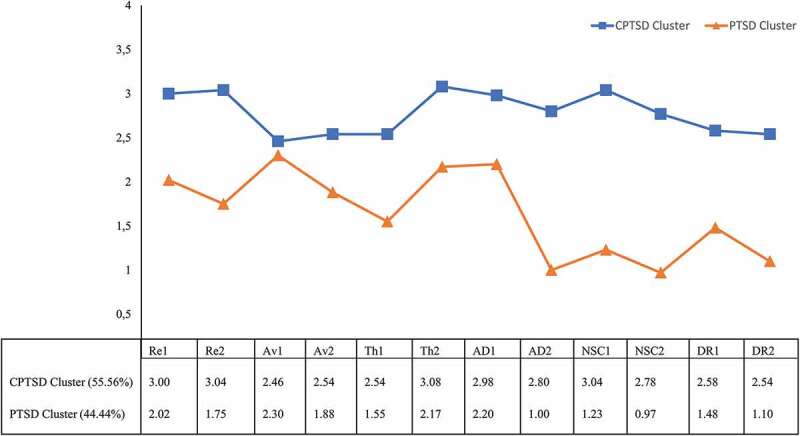
Symptom patterns of CPTSD by cluster

Note: Mean values of ITQ item responses according to most frequent cluster membership within all imputed data sets (*m*=20): upsetting dreams (Re1), reexperiencing the event in the here and now (Re2), avoidance of internal reminders (Av1), avoidance of external reminders (Av2), hypervigilance (Th1), jumpy/startled (Th2), long time to calm down (AD1), numb (AD2), failure (NSC1), worthless (NSC2), cut-off from others (DR1), difficulties to stay close to others (DR2).

Participants assigned to the first cluster (54.6%) reported higher average levels of symptoms across all items compared with participants in the second cluster (45.4%). Based on the symptom profiles (Figure 3), we labelled the clusters ‘CPTSD cluster’ and ‘PTSD cluster’. The highest scores in the PTSD cluster were found in the items assessing PTSD symptoms and the first item of affective dysregulation (long time to calm down). The mean scores of the item on avoidance of internal reminders yielded the smallest difference between the clusters. Individuals in the PTSD cluster reported lower levels of emotional numbing, negative self-concept, and difficulties in interpersonal relationships.

### 3.3. PMLD factors

Parallel analysis indicated a 4-factor solution across all PMLDC items. Specifically, the derived empirical eigenvalue for the fourth factor (1.99) was larger than its simulated counterpart (1.30), and the derived empirical eigenvalue for the fifth factor (1.47) was smaller than its simulated counterpart (1.73); hence, the 4-factor solution was accepted.

The factors corresponded to (1) discrimination & socioeconomic living conditions (DC), (2) language acquisition & barriers (LAB), (3) residence insecurity (RI), and (4) family concerns (FC). The 4-factor solution had the best fit to the data, accounting for 41.53% of the variance. Factor loadings are shown in Table 2. Since further investigation of the relationship to the CPTSD symptom clusters required factors that were as unambiguous as possible, the following items were deleted due to not loading adequately on their respective factors (≥ 0.3) or due to cross-loading with a difference lower than 0.2 as recommended (O’Connor, 2000) item no.: 3, 6, 7, 8, 9, 12, 15, 18, 20, 23, 25.

### 3.4. Characteristics of CPTSD cluster membership

The two clusters were compared in terms of the total number of childhood or adulthood PTE types and PMLD factors. MANOVA results showed differences in the two clusters [F = 2.35, *p* < .001, η^2^ = 0.15]. Individuals in the CPTSD cluster reported more problems language acquisition & barriers (t(85.13) = 2.81, *p* = .006). Overall, individuals in the CPTSD cluster reported a higher total number of childhood PTE types (t(85.72) = 2.22, *p* = .029). There were no differences in distress from the asylum process [t(85.44) = 1.40, *p* = .165, family concerns (t(85,34) = .41, *p* = .684) or discrimination & socioeconomical living conditions (t(84.78) = 1.90, *p* = .061). The clusters also did not differ in terms of reported adulthood PTE types (t(85.80) = .42, *p* = .673) or gender (χ^2^ = .01, *p* = .906). The overall results are presented in Table 3.

**Table 3.** Association with trauma & PMLDs

Predictor variable	CPTSD class M (SD)	PTSD class M (SD)	t(df)
Childhood PTE Types	7.54 (5.47)	5.06 (4.95)	2.26 (85.56)*
Adulthood PTE Types	8.26 (5.94)	7.74 (5.63)	0.41 (85.74)
**PMLDs 1** Socio-economical living conditions & Discrimination	11.34 (4.16)	9.68 (3.99)	1.87 (85.67)
**PMLDs 2** Language acquisition & barriers	17.10 (4.10)	14.45 (4.79)	2.78 (85.62)**
**PMLDs 3** Family concerns	13.53 (4.02)	13.18 (4.02)	0.35 (85.56)
**PMLDs 4** Residence insecurity	10.24 (3.80)	9.15 (3.50)	1.42 (85.69)

Note: * *p* < .05. ** *p* < .01. *** *p* < 0.001. Multiple imputation (*m* = 20) and *p*-value pooling was used.

## 4. Discussion

In the present study, we identified two homogeneous subgroups of CPTSD symptom presentation: a PTSD cluster and a CPTSD cluster (aim 1). We subsumed the multitude of PMLDs into four superior factors: discrimination & socio-economical living conditions, language acquisition & barriers, family concerns, and residence insecurity (aim 2). In a third step, we investigated the relationship between CPTSD cluster membership and demographic variables, childhood & adulthood PTE types, and the PMLD factors, and found associations with childhood PTE types, and the PMLD factors discrimination & economical living conditions and language acquisition & barriers.

The study reached a total of 93 Afghan treatment-seeking refugees and asylum seekers in Austria, with an unexpectedly high participation rate of women (45.2%). Almost half of the participants had a secure asylum status at the time of participation (granted asylum, Austrian citizenship). Consistent with other studies in treatment-seeking refugees, there was a high rate of individuals without a formal education (37.6%).

According to the first aim of the study, we investigated latent subgroups of CPTSD. The 2-cluster solution was identified as the best-fitting model, including a CPTSD cluster with a very high level across all symptoms and a PTSD cluster with increased PTSD symptoms and difficulties calming down. While the most commonly reported outcomes in studies examining CPTSD patterns were 3- and 4-subgroups (Brewin et al., 2017), one previous study in a treatment-seeking refugee sample identified the 2 subgroups as the best-fitting solution (Barbieri et al., 2019).

To address the second aim, the items of the PMLDC were subsumed into four factors, characterizing the domains of discrimination & socio-economic living conditions, family concerns, language acquisition & barriers, and residence insecurity. In previous studies, PMLDs associated with the asylum process and family concerns yielded separate factors (Laban et al., 2005; Silove, Steel, McGorry, & Mohan, 1998), while discrimination (Laban et al., 2005) and language (von Haumeder et al., 2019) were not consistently identified as separate domains. In contrast to Silove et al. (1998), discrimination and language resulted in two separate factors in our study, but discrimination formed a separate factor together with the frequently reported factor of socioeconomic life conditions (Laban et al., 2005).

Finally, in line with the third aim of the study, we examined the association of gender, traumatic experiences in childhood, discrimination & socio-economical living conditions, and difficulties in language acquisition & barriers with CPTSD cluster membership.

Consistent with previous studies (Cloitre et al., 2019; Palic et al., 2016), traumatic experiences in childhood were associated with the CPTSD cluster membership, whereas traumatic experiences in adulthood were not associated with the CPTSD cluster. Developmental theories emphasize that children and adults have to accomplish various developmental tasks at different time points. Traumatic experiences can hinder successful mastery of these tasks, such as the development of affect regulation skills, secure attachment, or a stable, positive self-concept (Cloitre et al., 2009). Due to the overlap of DSO with other mental disorders, we assume that the CPTSD class reflects a group of highly distressed individuals with further psychopathology. This might lead to an increased sensitivity to new stressors (Glaser, Van Os, Portegijs, & Myin-Germeys, 2006), interfere with the processing of prior traumatic experiences, or hamper the subsequent handling of PMLDs (Nickerson et al., 2015).

Our results indicate that not all forms of PMLDs are equally related to the CPTSD cluster: Only the PMLD factor language acquisition & barriers showed an association. Language is a crucial tool for adapting to a new environment and connecting with people. Language skills support the fulfilment of basic needs and adaptive coping with PMLDs, and foster the understanding of current situations (von Haumeder et al., 2019). Overall, few studies have examined the relationship between language skills and trauma sequelae. Preliminary results showed that difficulties in acquiring the host language mediated the effect between traumatic experiences and PTSD (Kartal et al., 2019).

While our results do not allow for conclusions regarding directionality, we assume a bidirectional relationship between CPTSD symptoms and the associated PMLD factor, language acquisition & barriers. A reciprocal influence between CPTSD symptoms and PMLD factors could lead to a downward spiral of elevated CPTSD symptoms, increased distress caused by PMLDs, and greater difficulties in handling the mental disorder and PMLDs. For example, difficulties in language acquisition, language barriers, and CPTSD symptoms such as associated affective dysregulation, re-experiencing symptoms and deficits in social skills might not only increase the risk of experiencing overwhelming situations but also reduce the possibilities to actively cope with those, which in turn could lead to increased emotional distress. Affective dysregulation might result in a more intense response to emotional cues and increased difficulty in calming down. In the long term, these experiences of difficulties in language acquisition and repeated confrontation with language barriers as well as associated dependency on others, might lead to increased CPTSD symptomatology, such as further deterioration of the negative self-concept, more difficulties in relationships, or social withdrawal. These dynamics and cognitive deficits associated with CPTSD (Etkin, Gyurak, & O’Hara, 2013) may further complicate language acquisition. In turn, the resulting problems in everyday life may increase the risk of social exclusion, and aggravate mental health problems (Kartal et al., 2019). Additionally, low trust in the health care system can impede access to health care services (Ben, Cormack, Harris, & Paradies, 2017).

### 4.1. Strengths and limitations

The major strength of the present study lies in the investigation of the research questions in an extremely psychologically strained, hard-to-reach population of refugees and asylum seekers. The face-to-face, interpreter-assisted, fully structured interview conducted by a trained clinical psychologist allowed for the participation of illiterate individuals and enabled the implementation of preventive interventions to reduce stress and/or concentration problems during the assessment. Moreover, the person-centred approach enabled us to examine homogeneous subgroups of CPTSD symptoms and their different associations with various PMLD factors.

Nevertheless, some limitations of the study should be noted. All subjects in our sample were Afghan refugees in Austria and it cannot be excluded that different findings would have emerged in other ethnic groups or cultural contexts. Due to the cross-sectional study design, it is not possible to establish directionality. Although special attention was given to the selection of measures, and statistical methods suitable for small samples, the small sample size and the selection of a widely used but insufficiently evaluated test instrument without established subscales to assess PMLDs could still reduce the statistical power and increased the likelihood of a Type II error. It should also be noted that the study was not preregistered and Covid-19 pandemic interrupted the assessment process. As daily activities and social contacts have been limited in recent months, this might have influenced symptom severity and the experience of PMLDs. However, given the strengths of our study, the results mirrored the psychological distress in treatment-seeking traumatized refugees and asylum seekers and its association with PMLDs. Thus, future post-pandemic replications of the study with larger samples and a longitudinal design are recommended to further investigate the generalizability and the directionality of the results.

### 4.2. Clinical implications

The present findings underline the importance of understanding mental health problems while taking into account traumatic experiences and PMLDs (Nickerson, Bryant, Silove, & Steel, 2011). Childhood PTE types and PMLDs in the form of difficulties in language acquisition and language barriers, should be especially considered in individuals with high CPTSD symptomatology and integrated into diagnostic and treatment strategies. A reduction of the psychological strain associated with PMLDs could be achieved by promoting, for example, affect regulation, social skills, and a positive self-concept, which are acknowledged elements of the psychological treatment of CPTSD (Schäfer et al., 2019). Trauma-focused treatment strategies could reduce potential re-experiencing symptomatology. Individual difficulties in language acquisition and distress due to language barriers should be reduced by personalized psychological interventions to counter the particular problem, such as stress management to improve cognitive deficits associated with CPTSD. The establishment of trauma-sensitive language courses might be a further interdisciplinary task for pedagogues and psychologists (Kartal et al., 2019).

### 4.3. Conclusion

Our findings highlight that different CPTSD symptom clusters are associated to varying degrees with different trauma histories and forms of PMLDs. Programmes addressing the mental health needs of refugees and asylum seekers should include interventions at various levels, including psychological therapy, trauma-sensitive language courses, and measures to facilitate inclusion in the host society.

## Data Availability

The data that supports the findings of this study are openly available in ‘Zenodo’ at https://doi.org/10.5281/zenodo.5054032. Further information and R Scripts are available from the corresponding author upon reasonable request.
